# Mid-infrared femtosecond laser-induced damages in As_2_S_3_ and As_2_Se_3_ chalcogenide glasses

**DOI:** 10.1038/s41598-017-06592-3

**Published:** 2017-07-26

**Authors:** Chenyang You, Shixun Dai, Peiqing Zhang, Yinsheng Xu, Yingying Wang, Dong Xu, Rongping Wang

**Affiliations:** 1 Laboratory of Infrared Materials and Devices, The Research Institute of Advanced Technologies, Ningbo University, Ningbo, 315211 China; 2 Key Laboratory of Photoelectric Detection Materials and Devices of Zhejiang Province, Ningbo University, Ningbo, 315211 China

## Abstract

In this paper, we report the first measurements of mid-infrared (MIR) femtosecond laser-induced damage in two typical chalcogenide glasses, As_2_S_3_ and As_2_Se_3_. Damage mechanism is studied via optical microscopy, scanning electron microscopy and elemental analysis. By irradiating at 3, 4 and 5 μm with 150 fs ultrashort pulses, the evolution of crater features is presented with increasing laser fluence. The dependence of laser damage on the bandgap and wavelength is investigated and finally the laser-induced damage thresholds (LIDTs) of As_2_S_3_ and As_2_Se_3_ at 3 and 4 μm are calculated from the experimental data. The results may be a useful for chalcogenide glasses (ChGs) applied in large laser instruments to prevent optical damage.

## Introduction

ChGs have attracted intensive research interests in past decades due to their low phonon energies, high linear and nonlinear refractive indexes and wide transparency in the infrared region. They have shown great potentials for applications in biosensors^1^, atmosphere pollution monitoring^2^, frequency metrology^3^, and temperature sensors^4^. As a matter of fact, waveguide or fiber based devices have been explored based on high nonlinearity of ChGs, for example, mid-infrared laser sources like supercontinuum generation, and high speed signals processor using chalcogenide waveguide-based devices. However, one of the drawbacks of ChGs is their relatively weak mechanical properties. Especially, ChGs usually underwent catastrophic damage while exposed to high intensity laser beams. It has also been demonstrated that, as the Se was progressively replaced by S, the damage threshold rose and the tendency for the surface to “burn” disappeared^5^. P. Hari *et al*. firstly studied the ablation of bulk amorphous As_2_Se_3_ with picosecond laser pulses and they noted a sharp increase in ablation threshold with the decrease in the macropulse length^6^. Zhang *et al*. reported that the damage threshold increased with increasing Ge concentration in Ge-As-S glasses irradiated by femtosecond laser pulses at an average power of 33 mW^7^. However, a rigorous investigation on the laser damage threshold of ChGs in the femtosecond regime is still absent.

Emerging applications of ChGs require the understanding of the interaction between laser and materials especially in the mid-infrared region. For example, increasing incident laser power can increase the intensity of the SC generation in chalcogenide waveguide and fiber, and an enhancement of the sensitivity can be achieved in chalcogenide based mid infrared sensors. Once a threshold was exceeded, the glass surface “burnt” and evaporated creating gross craters far bigger than the irradiated spot. Therefore, for practical applications, it is important to investigate the laser damage on the materials especially in mid infrared region.

Several kinds of interactions between materials and lasers, including ablation and damage, have been proposed when transparent media are radiated by high power femtosecond lasers^8–13^. Unlike optical damage caused by nanosecond pulses or longer pulses, the damage mechanism of the femtosecond laser is mainly due to accumulation of conduction band electrons (CBEs) rather than thermal accumulation^11^. Due to extremely short pulse width, the laser energy is absorbed by electrons faster than it is transferred to the lattice, and this suppresses the thermal conduction and makes cold mechanism possible. The laser damage mainly consists of three processes^8^. Firstly, photoionization (PI) causes the excitation of the electrons from the valence to the conduction band. Then, these initial CBEs, as the seed electrons, motivate avalanche ionization (AI) process further to generate more CBEs. Finally, numerous CBEs accumulate rapidly to form dense plasma. Once the energy of the plasma exceeds the threshold of the media, irreversible optical damage occurs on the surface of the media^10, 14^. Moreover, the damage processes are affected by material properties, pulse duration, number of pulses, sample surface state, and especially laser wavelength. In addition, a majority of femtosecond laser damage studies on transparent media published to date were performed with the light pulses centered around the near-infrared (NIR) wavelength of Ti:sapphire and dye lasers. For example, Borowiec *et al*. reported the single pulse femtosecond laser damage threshold of indium phosphide at the widest wavelength range from 400 to 2050 nm^15^, D.M.Simanovskii *et al*. presented laser-induced damage studies at a wavelength of 8000 nm^16^. However, up to now, there is almost no research of femtosecond laser-induced damage threshold of ChGs in the mid-infrared (MIR) region. Compared with NIR wavelengths, the photon energy in MIR is lower and more photons are required to excite the electrons. Hence, with increasing wavelength, the process of multi-photon ionization (MPI) is the crucial factor of the laser induced damage^17^.

In the paper, we systematically investigated laser damage of two typical chalcogenides, As_2_S_3_ and As_2_Se_3_ glasses, induced by mid-infrared pulses. Using an OPA system delivering ~150 fs pulses, the effects of the laser intensity, wavelengths (3/4/5 μm), and band gap of the glasses on laser damage were investigated using different techniques including the super long depth of view optical microscope and scanning electron microscope (SEM). The results can be useful for the design of MIR supercontinumm sources with high output powers based on chalcogenides fiber. In addition, LIDTs of As_2_S_3_ and As_2_Se_3_ at different wavelengths are essential to avoid contamination in the real applications since they contain poisonous element of Arsenic.

## Results and Discussion

### Optical tests of samples

To avoid the effect of the sample surface quality on our experiments, all the sample were optically polished with a root-mean-square (RMS) surface roughness of 3 ± 0.6 nm and a peak-to-valley surface flatness of 50 ± 5.0 nm, respectively, measured by interferometer. Each sample has a thickness of ~2.0 mm.

**Figure 1:**
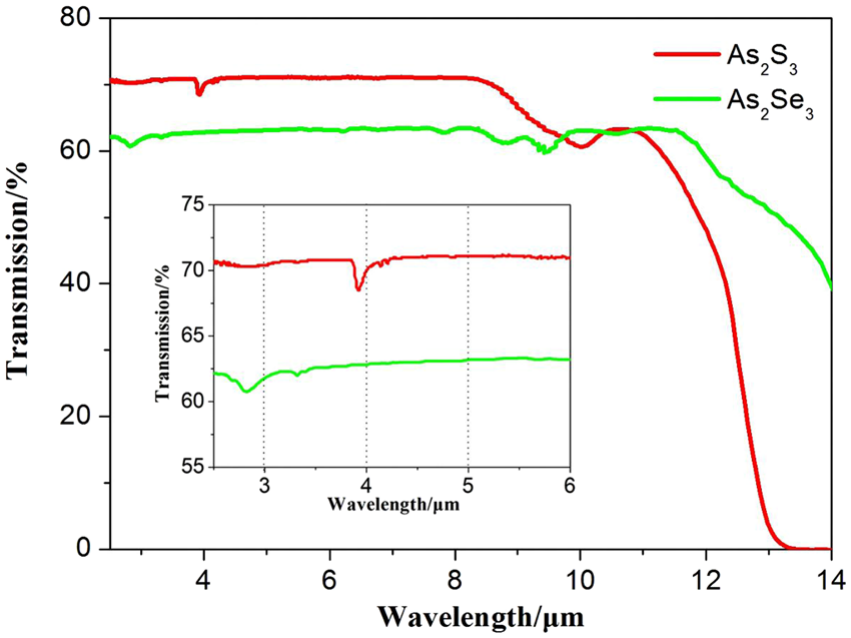
Transmission spectra of As_2_S_3_, the red line, and As_2_Se_3_, the green line. The inset shows the spectra from 2.5 to 6 μm.

IR transmission spectra of As_2_Se_3_ and As_2_S_3_ glasses as shown in Fig. 1 were measured using a Fourier transform infrared spectroscopy (FTIR) (Thermo Scientific, Nicolet 380, USA) at a range from 2.5 μm to 15.0 μm at room temperature. From the inset of Fig. 1, for our high-purity samples, only some weak impurity absorption bands were detected, which were ascribed to the vibration of the H-O bonds at 2.79 µm for As_2_Se_3_ and S–H bonds at 3.83 µm for As_2_S_3_. These broad absorption bands could have a slight effect on the damage threshold of the samples irradiated at 3 µm and 4000 µm. It is well documented that the Tauc gap of As_2_S_3_ and As_2_Se_3_ glasses are 2.4 eV^18^ and 1.7 eV^6^. Therefore, it is essential to have the 6th, 8th and 10th order optical process to excite the electrons across the bandgap for As_2_S_3_, and the 4th, 6th, and 7th order optical process for As_2_Se_3_, using 3 μm, 4 μm and 5 μm irradiation wavelength, respectively.

### The damage evolution with laser fluence

**Figure 2:**
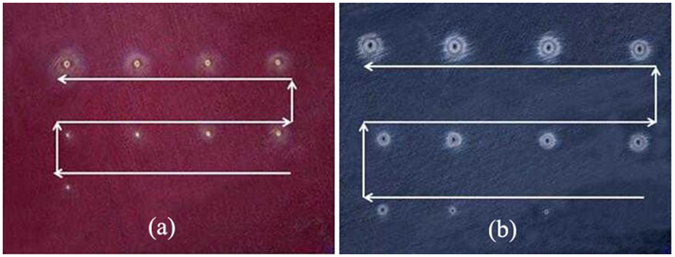
Laser-induced damage arrays after irradiation with a 3 μm femtosecond laser on the 2 mm thickness samples: (**a**) As_2_S_3_ glass; (**b**) As_2_Se_3_ glass. (Laser average power increasing as arrows shows).

In our experiments, laser-induced damage was performed in the “S-on-1” regime according to ISO standard 11254-1.2. Figure 2(a) and (b) show optical images of arrays of structures on the surfaces of As_2_S_3_ and As_2_Se_3_ glasses, respectively, produced by multi laser pulses (60000 pulses). We gradually increase the average laser power from 2.5 mW to 30 mW with a step of 2.5 mW (44.2 mJ/cm^2^). Obviously, we observed that the damage crater area expanded and depth increased with increasing laser intensity. The value of the average laser power that begins to induce observable modification on the surface is defined as the critical powers (P_cr_) in our work. Hence, the intensity threshold I_th_ for the glasses can be calculated as

1 $${I}_{th}=\frac{{P}_{cr}}{R\tau \pi {D}^{2}/4}$$

where D is the diameter of the beam spot, R is repetition rate and τ is pulse duration. The I_th_ was further transformed into laser fluence threshold F_th._

2 $${F}_{th}=\frac{2{P}_{cr}}{R\pi {D}^{2}/4}$$

**Figure 3:**
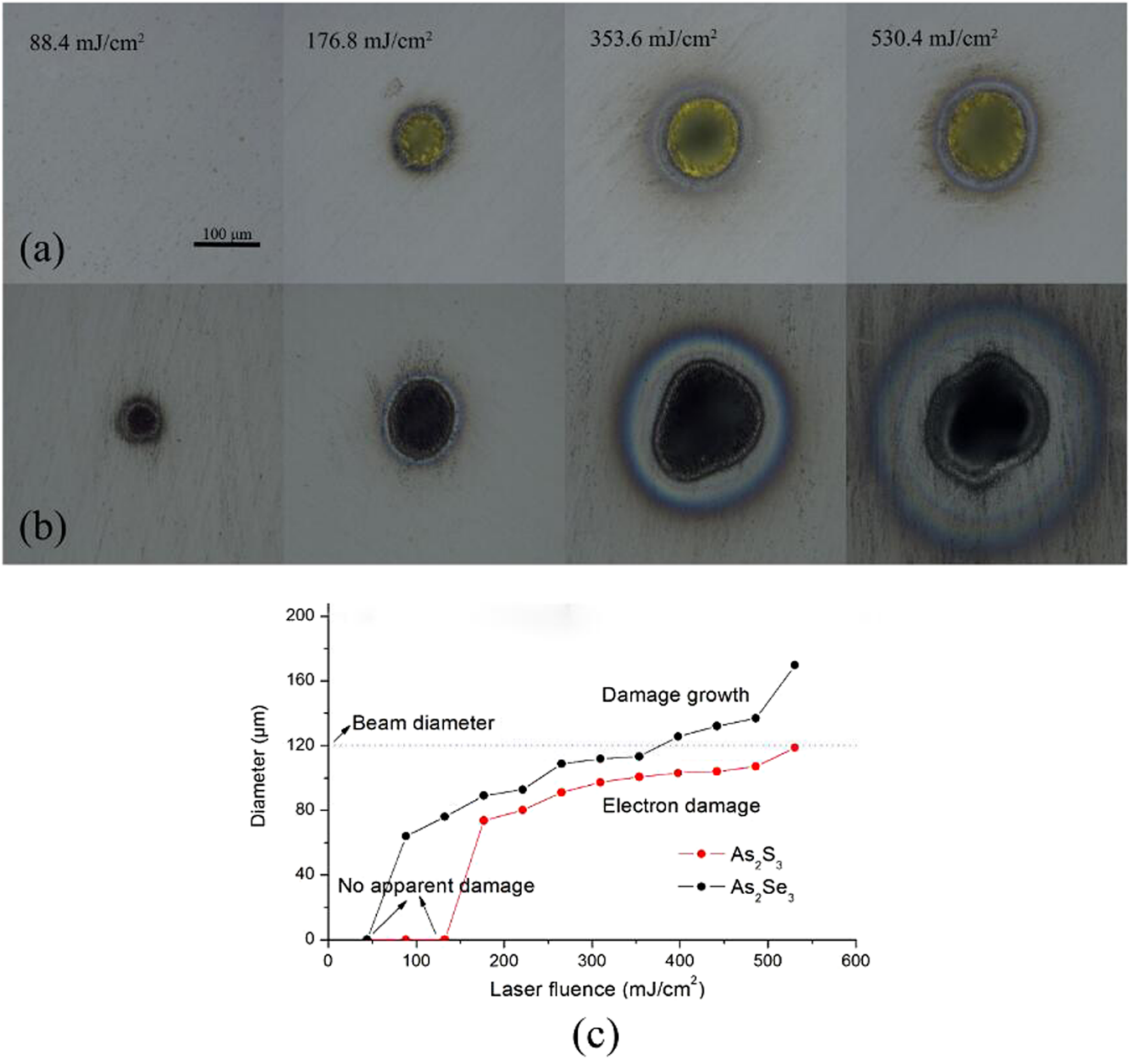
Optical micrographs of multi pulses damaged As_2_S_3_ (**a**) and As_2_Se_3_ (**b**) at a range of laser fluences. (**c**) The diameter of damage crater observed by optical microscope as a function of laser fluence.

After As_2_S_3_ and As_2_Se_3_ samples were irradiated by 3 μm laser pulses, we observed damage spots by optical microscope to investigate the dependence of the damage on the laser intensity. Figure 3(a),(b) show the microscope images of As_2_S_3_ and As_2_Se_3_ glasses damaged under laser irradiation with different laser fluence. Once the laser power exceeded P_cr_ of the sample, the damage crater occurred and expanded with increasing power, and thus with increasing laser fluence. Compared with that of As_2_S_3_ glass, the damage of As_2_Se_3_ glass appear at lower laser fluence and the diameter of the damage center area for As_2_Se_3_ sample is always bigger than that for As_2_S_3_ sample at the same laser fluence. Obviously, A_2_S_3_ glass has a higher laser damage threshold since an electron can only be excited from the valence band to the conduction band via the 6th order optical process in As_2_S_3_ glass at 3 μm during the MPI process while this can be done via the 4th order optical process in As_2_Se_3_. In Fig. 3(c), when laser fluence exceeded 353.6 mJ/cm^2^, the damage crater diameter of As_2_Se_3_ started growing larger than beam diameter as shown in the third images in Fig. 3(a) and (b) from the left. We deduced the damage growth was caused by the “second” damage that co-existed with of other damage mechanisms rather than only electron accumulation, which is in agreement with the formation of “crater-in-crater” shape presented by Inam Mirza *et al*.^19^.

**Figure 4:**
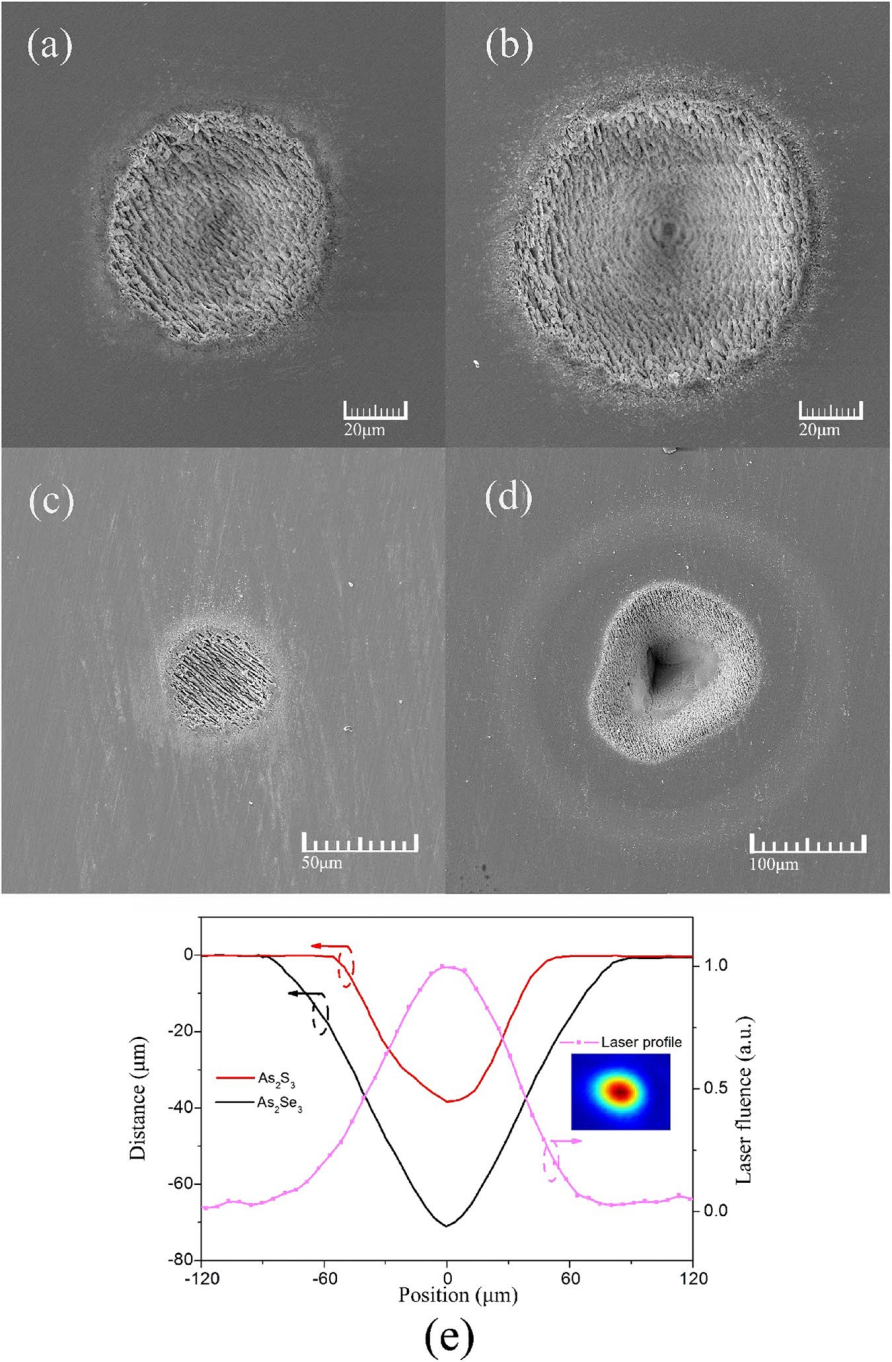
SEM micrographs of multi pulses damage spots at 3 μm. The damage spots at *P* = *P*_*cr*_ = 10 mW (**a**) and *P* = 30 mW (**b**) for As_2_S_3_, and at *P* = *P*_*cr*_ = 5 mW (**c**) and at *P* = 30 mW (**d**) for As_2_Se_3_. (**e**) The beam intensity profile and the depth profiles of the damage craters at average laser power of 30 mW.

In order to further research the damage process, we observed the damage spots using SEM, and more detailed crater features were shown in Fig. 4. At a low laser fluence (P = P_cr_), the damage features can present initial electron damages in Fig. 4(a) and (c). The surface of the crater was full of the rough ripples without melting. These ripples are unique in the sample with multiple pulses irradiation onto the same spot. S.H. Messaddeq *et al*. first observed and studied the laser-induced periodic surface structures (LIPSS) in Ge-S based chalcogenide glasses irradiated by femtosecond laser (1 kHz, 34 fs, 806 nm)^20^. At a higher laser fluence (P = 30 mW) in Fig. 4(b) and (d), we observed that the crater has different morphological features, which may be caused by ablation, central modification, re-deposited materials and outer modification, from center to outer^21^. This in turn is due to the Gaussian distribution of the laser beam intensity Such the beam intensity profile and the correponding depth profiles of damage craters of As_2_S_3_ and As_2_Se_3_ samples also are shown in Fig. 4(e). It is clear that the strongest laser power causes the maximum damage.

**Figure 5:**
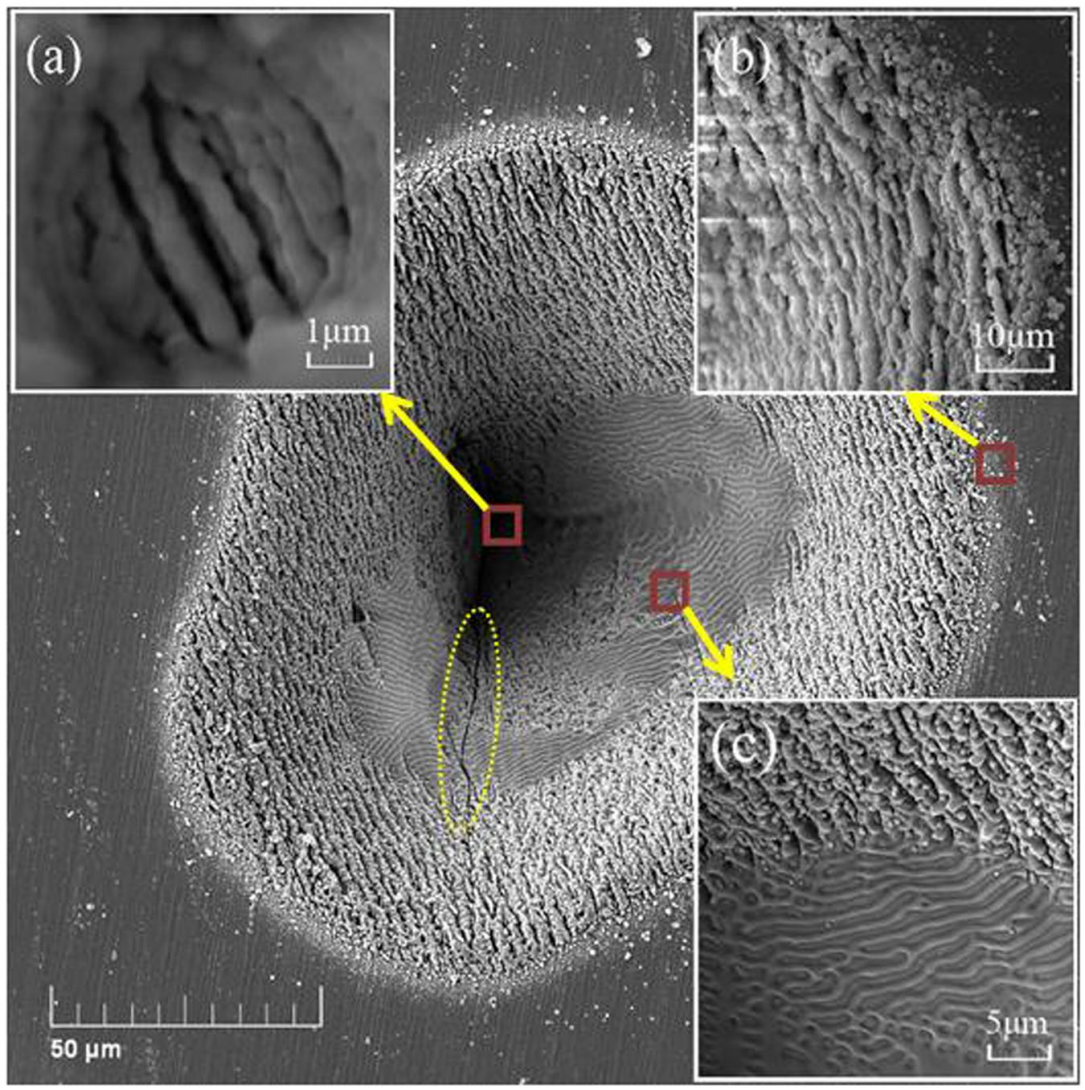
High resolution SEM micrograph of As_2_Se_3_ damage crater at 30 mW. The inset image (**a**) shows boundary between melting and electron damage. The inset image (**b**) shows edge of electron damage. The inset image (**c**) shows bottom of damage crater. A crack is marked in yellow dotted line circle.

A higher resolution SEM micrograph is given in Fig. 5 for As_2_Se_3_ sample under high laser fluence irradiation. The insets images (a), (b) and (c) in Fig. 5 show the bottom of the damage crater, the electron damage edge, and the melting edge, respectively. Obviously, two types of damage feature can be observed in the inset image (c). The rough electron damage feature is on the top part of the crater, however, smooth melting morphology gradually appears on the bottom part of the crater. In addition, we also observed some micro cracks on the main panel of Fig. 5 as shown by the yellow dot line oval, and this may be due to thermal stress during laser irradiation. We believe that, the PI process is dominated during the initial surface damage. Such a process can produce numbers of the defects on the surface, which can strengthen material thermal accumulation under ultrahigh repetition laser pulses. Then, the PI process no longer plays the major role in subsequent damage growth.

### LIDTs measurements at different wavelengths

**Figure 6:**
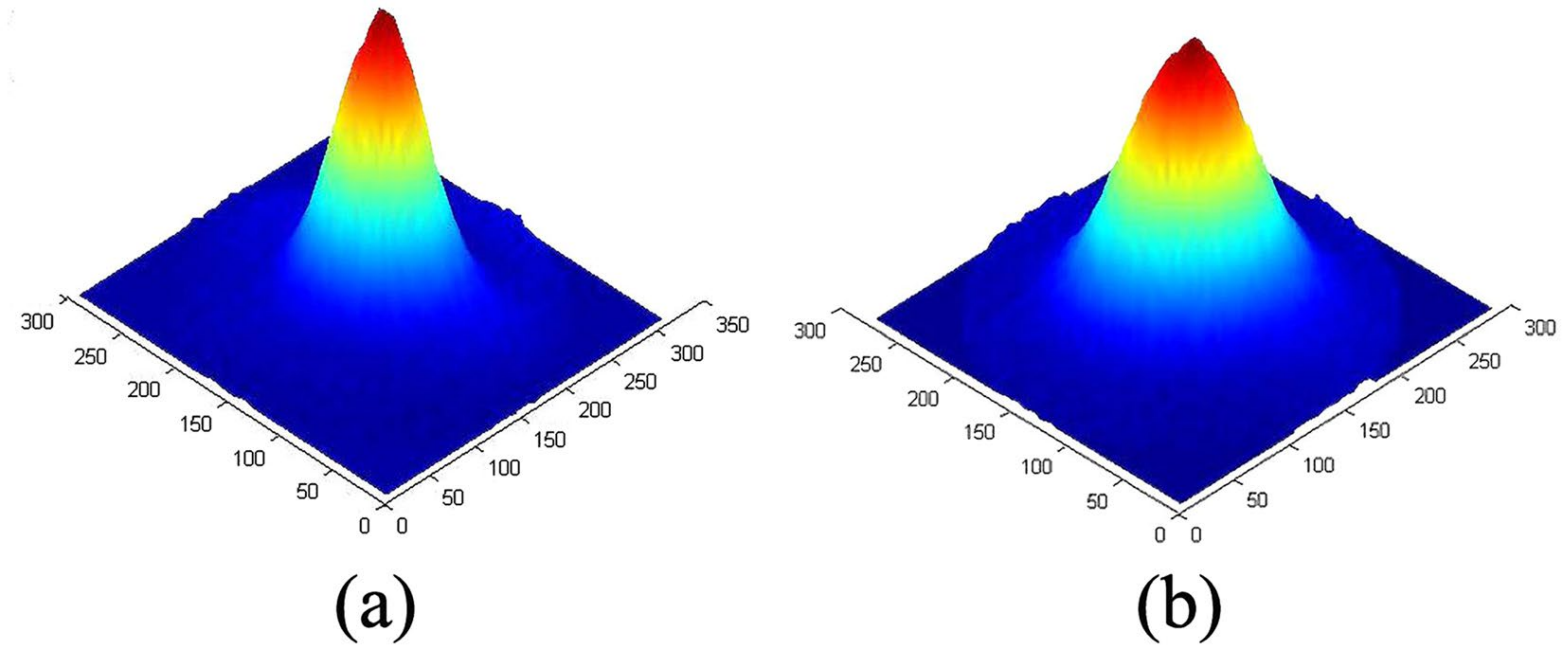
3D images of beam intensity profile at 3 μm (**a**) and at 4 μm (**b**).

To further understand the laser wavelength on the LIDTs, the same laser damage experiments were performed with the same condition at 4 μm. Obviously, more photons are required to excite the electrons for 4 μm light with a lower photon energy (about 0.31 eV) Fig. 6(a) and (b) show 3D images of the beam intensity profile at 3 μm and 4 μm, respectively. It was found that, the beam profiles of 3 μm and 4 μm at the same average beam intensity exhibit similar Gaussian distribution, expect for slightly higher relative intensity in the center of the beam spot at 3 μm.

**Figure 7:**
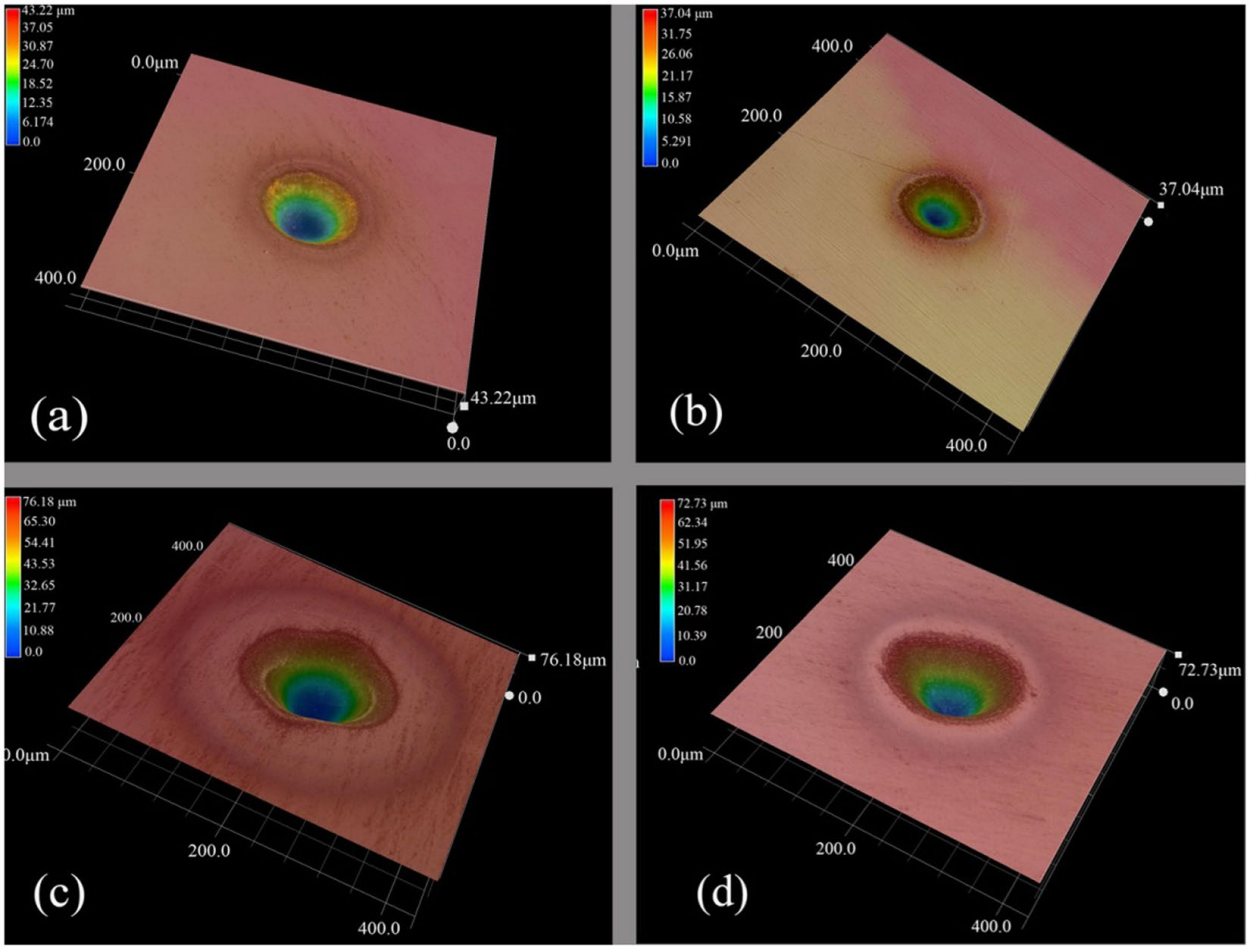
3D Optical micrographs of laser-induced damage sites after irradiation with average power of 30 mW at 3 μm: (**a**) As_2_S_3_ glasses; (**c**) As_2_Se_3_ glasses, and at 4 μm: (**b**) As_2_S_3_ glasses; (**d**) As_2_Se_3_ glasses.

Figure 7 shows the comparison of the damage spots under 3 μm and 4 μm irradiation with the same laser power. It was found that, both As_2_S_3_ and As_2_Se_3_ had less damage at 4 μm than at 3 μm, and this was reflected by smaller diameter and depth of the craters. Using the same method to measure P_cr_, we obtained P_cr_ = 17.5 mW for As_2_S_3_ and P_cr_ = 15 mW for As_2_Se_3_ at 4 μm. To compare LIDT at 3 μm and 4 μm quantitatively, the related parameters were listed in Table 1, from which it is clear that As_2_S_3_ exhibits better laser damage resistance than As_2_Se_3_ at the same wavelength. For different wavelengths, the process of MPI requires 6 photons at 3 μm and 8 photons at 4 μm for As_2_S_3_, while the 4 photons at 3 μm and 6 photons happens at 4 μm for As_2_Se_3_. For As_2_S_3_ glass, P_cr_ is about 10 mW at 3 μm and 17.5 mW at 4 μm, and thus LIDT is 176.8 mJ/cm^2^ at 3 µm and 309.5 mJ/cm^2^ at 4 µm. For As_2_Se_3_ glass, P_cr_ is about 5 mW at 3 μm and 15 mW at 4 μm, and thus LIDT is 88.4 mJ/cm^2^ at 3 µm and 265.3 mJ/cm^2^ at 4 μm. We determined the spatial period from SEM images and the results were listed in column 3 of Table 1. M. Rohloff *et al*.^22^ and Jorn Bose *et al*.^23^ proposed that a saturation value of the spatial period of the surface structure, Λ_LIPSS_, can be estimated as ∼λ/n, where λ is the laser irradiation wavelength, and n is the refractive index of the glass. The calculated spatial period was thus determined, and the results were listed in the column 4 of Table 1 (with refractive index n(As_2_S_3_) = 2.43 and n(As_2_Se_3_) = 2.78 at 3 μm, and n(As_2_S_3_) = 2.42 and n(As_2_Se_3_) = 2.77 at 4 μm.). It is clear that, all the experimental values (in the column 3) are in agreement with the corresponding calculated spatial period.

**Table 1:** Λ_LIPSS_ and LDIT of As_2_S_3_ and As_2_Se_3_ glasses with the 150 fs laser at different wavelength.

Glass	Wavelength	Λ_LIPSS_	λ/n	p_cr_	LDIT
As_2_S_3_	3 μm	880~1230 nm	1235 nm	10 mW	176.8 mJ/cm^2^
	4 μm	920~1500 nm	1653 nm	17.5 mW	309.5 mJ/cm^2^
As_2_Se_3_	3 μm	750~1070 nm	1079 nm	5 mW	88.4 mJ/cm^2^
	4 μm	1050~1380 nm	1444 nm	15 mW	265.3 mJ/cm^2^

We also investigate the laser damage features of As_2_S_3_ and As_2_Se_3_ glasses at 5 μm, the process of MPI requires 11 photons and 8. We did not observe any apparent surface modifications at any laser power available. Moreover, even we irradiated the samples with more times than 60000 pulses, there is still no damage. We deduce that the dynamic balance between the accumulation and the loss of the electrons in the conduction band suppresses the initial laser damage of samples. This result also demonstrates that the damage is initiated by MPI process rather than thermal accumulation.

### The elements variation analysis

**Figure 8:**
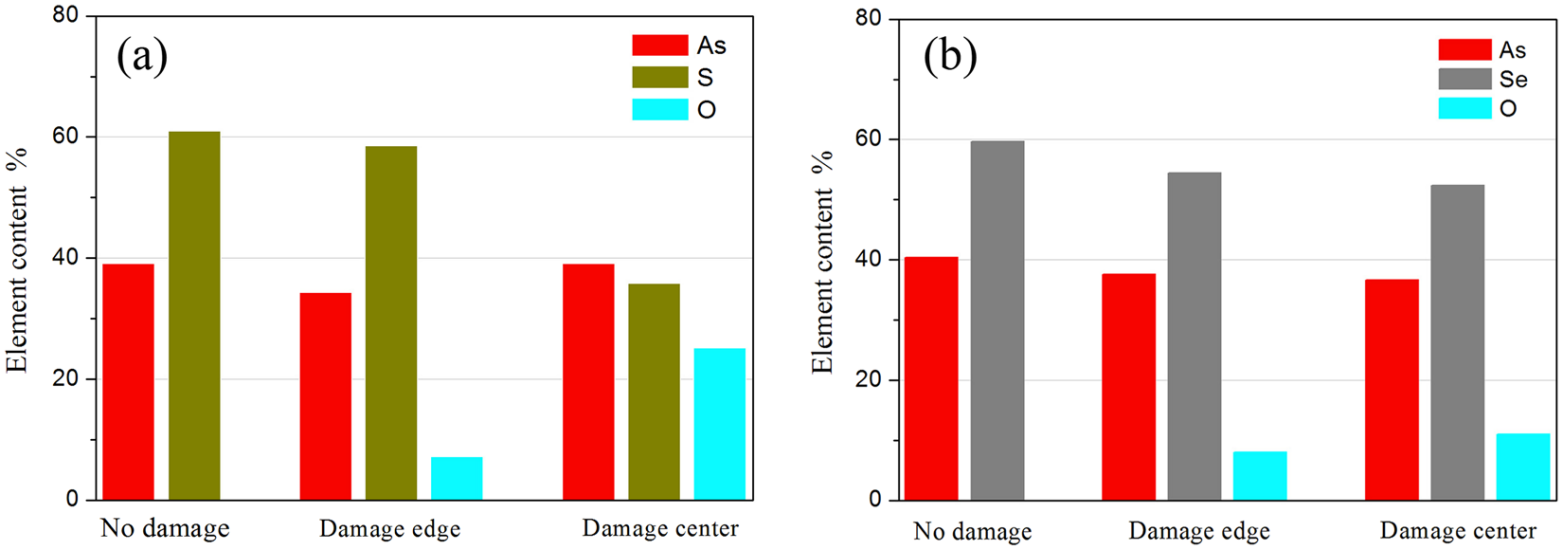
(**a**) Elemental compositions of As_2_S_3_; (**b**) Elemental compositions of As_2_Se_3_. The samples were damaged at 3 μm as an example.

We also analyzed the change of the compositions on the surface measured by an energy dispersive x-ray spectroscopy. From Fig. 8(a) and (b) show obvious change of the chemical compositions for the glasses before and after irradiation, and the relative measurement error is about ±0.5%. In Fig. 8(a), the glass without irradiation shows a good As/S ratio of 2/3. However, As/S increases from about 2/3 to even ≥1 with increasing laser power from the edge to the center. Meanwhile, oxygen appears in the glass with irradiation. Obviously, the structural units of As_2_S_3_ is broken apart into individual ions under irradiation and then easily recombined with oxygen, and the decomposed S is easily volatilized, leading to increasing As/S ratio. On the other hand, although As_2_Se_3_ is easily damaged by high power laser and more material is removed, the ratio As/Se in Fig. 8(b) show relatively small change due to less volatilization of Se compared with S. As_2_S_3_ glass surface is covered by more crystals after irradiation resulting in a rougher surface of the crater.

## Conclusion

We investigated laser fluence and wavelength dependence of damage features in bulk As_2_S_3_ and As_2_Se_3_ glasses under multiple MIR femtosecond pulses. Damage mechanisms are studied using optical microscopy, scanning electron microscopy and elemental analysis. With different irradiation wavelength of 3, 4 and 5 μm, the evolution of the damage becomes significant with increasing laser fluence, and the features of “initial” and “secondary” damage are studied experimentally for 150-fs 60000-pulses. LIDT of As_2_S_3_ and As_2_Se_3_ at 3 and 4 μm are calculated from the experimental data. Based on the results, MIR laser-induced damage of ChGs was concluded to be firstly initiated by the accumulation of electrons and subsequently driven by thermal accumulation. Generally, As_2_S_3_ glass shows better laser damage resistance than As_2_Se_3_ glass due to its wider band gap. The results are helpful for the use of these chalcogenide glasses in various optical systems under high laser power.

## Method

### Glass preparation

High-purity 6N (99.999999%) chemical elements of arsenic, selenium, and sulfur were used as starting materials to prepare As_2_S_3_ and As_2_Se_3_ glasses. The raw materials were then placed in the silica ampoule, and the standard purification procedures were employed to further purify the starting materials. Then, the sealed silica ampoule was kept at 800 °C for 12 h in a rocking furnace to ensure homogeneity and then quenched in water to avoid crystallization. The as-prepared glass rods were further annealed at 30 °C below transformation temperature Tg for 3 h to minimize internal stress and then cooled to room temperature.

### The experimental setup

**Figure 9:**
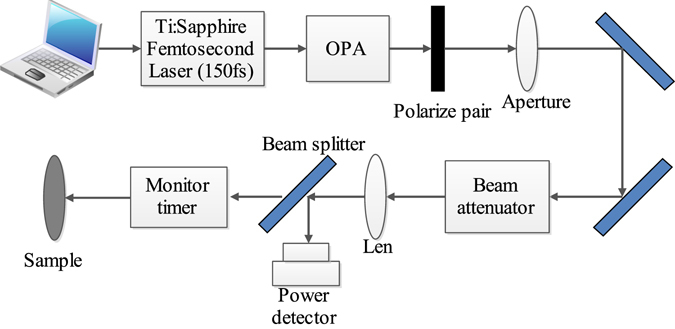
Schematic of the experimental configuration (drawn by the software of Edraw Max).

The experimental setup of laser-induced damage is presented in Fig. 9. A Ti:Sapphire femtosecond laser (Coherent, “Mira900D+”) combined with an optical parametric amplifier-OPA (Coherent, “Legend Elite + OperA Solo”) produces 150 fs pulses at a maximum repetition rate of 1 kHz. The wavelength can be tuned from 2 μm to ~20 μm. The laser beam was focused onto the front surface of the samples with a diameter of D ≈ 120 μm (defined as 1/e^2^ of peak intensity) by a lens of CaF_2_ with a 75 mm focal length. We adjusted the distance between the lens and sample to tune the focal spot size by a three-dimensional adjustable frame, and thus the focal spot size was kept almost similar when we changed the wavelength. We also used an infrared beam profiler (Ophir, “PY-III-HR-C-A”) to get high quality laser beam. Due to the Gaussian distribution of the laser beam as shown in Fig. 6, we calculated the peak fluence F_peak_ with^14^:

3 $${F}_{peak}=\frac{2{E}_{pulse}}{\pi {D}^{2}/4}=\frac{2{P}_{avg}}{R\pi {D}^{2}/4}$$

In our experiments, the samples were irradiated at 3, 4 and 5 μm central wavelength with 60000 pulses with the laser fluence from 44.2 mJ/cm^2^ to 530.5 mJ/cm^2^ per pulse.

### Analysis of crater morphology

After laser irradiating, all multi-pulses craters on the surface were observed by a super long depth of view optical microscope (Keyence, JAPAN, “VEX-1000E”) and more detailed characterization of crater morphology and elemental analysis was analyzed by SEM (Tescan, CZECH, and “VEGA3SB-EasyProbe”) equipped with energy dispersive x-ray spectroscopy(EDX).
